# Two-year follow-up after drug desensitization in mucopolysaccharidosis

**DOI:** 10.1186/s13023-024-03516-z

**Published:** 2024-12-27

**Authors:** Federico Spataro, Roberto Ria, Nada Chaoul, Antonio Giovanni Solimando, Vanessa Desantis, Angelo Vacca, Danilo Di Bona, Attilio Di Girolamo, Luigi Macchia

**Affiliations:** 1 Post Graduate School in Allergology and Internal Medicine “Guido Baccelli”, Department of Precision and Regenerative Medicine and Ionian Area—(DiMePRe-J), School of Medicine, Aldo Moro University of Bari, Bari, 70124 Italy; 2 Guido Baccelli Unit of Internal Medicine, Department of Precision and Regenerative Medicine and Ionian Area—(DiMePRe-J), School of Medicine, Aldo Moro University of Bari, Bari, 70124 Italy; 3 Division of Medical Oncology, A.O.U. Consorziale Policlinico di Bari, Bari, Italy; 4 Department of Precision and Regenerative Medicine and Ionian Area (DiMePRe-J), Section of Pharmacology, University of Bari “Aldo Moro” Medical School, Bari, Italy; 5 Internal Medicine, Liver Unit, Department of Medical and Surgical Sciences, C.U.R.E. (University Centre for Liver Disease Research and Treatment), University of Foggia, 71122 Foggia, Italy

**Keywords:** Mucopolysaccharidosis, Enzyme replacement therapy, Scheie syndrome, Hunter syndrome, Drug allergy, Desensitization, Omalizumab, Lysosomal storage disorders

## Abstract

**Background:**

Mucopolysaccharidosis (MPS) type 1 S and type 2 are rare lysosomal storage disorders characterized by impaired enzyme production, resulting in glycosaminoglycans accumulation within lysosomes. Enzyme Replacement Therapy (ERT) with laronidase and idursulfase are first line treatments, respectively. However, infusion-related hypersensitivity reactions (HR) may lead to ERT discontinuation. Thus, desensitization can be performed to restore ERT.

**Methods:**

We report on a two-year follow-up after a combined desensitization approach in two MPS patients experiencing HR to ERT. This approach consists of intravenous rapid desensitization combined with the subcutaneous allergen immunotherapy-like desensitization with the culprit recombinant enzyme.

**Results:**

The first patient, suffering from MPS type I, underwent to the combined desensitization approach, and subsequently tolerated weekly standard laronidase infusions for 13 months when HR occurred again. Then, a monthly omalizumab (anti-IgE monoclonal antibody) administration was implemented allowing the patient to restore ERT. The second patient, diagnosed with MPS type 2, was subjected to a similar combined desensitization strategy with idursulfase, and achieved a total desensitization after one year, confirmed by negative skin tests. Thus, he continued standard ERT infusions without HR occurrence.

**Conclusion:**

The combined desensitization approach proved effective in conferring immunotolerance for at least one year in both MPS patients, also demonstrated by the negative skin tests in one patient. However, when immunotolerance to ERT is lost, omalizumab administration can be a valid option in restoring ERT.

**Supplementary Information:**

The online version contains supplementary material available at 10.1186/s13023-024-03516-z.

## Letter to the editor

To the editor,

Mucopolysaccharidosis (MPS) type 1 S and type 2 are rare lysosomal storage diseases caused by impaired enzyme production, leading to glycosaminoglycan accumulation within lysosomes. In MPS type 1 S, reduced function of the alpha-L-iduronidase gene occurs, while MPS type 2 is characterized by a mutation in the iduronate-2-sulfatase gene [1]. Enzyme replacement therapy (ERT) is the only available therapeutic approach for these patients which consist of the weekly intravenous administration of the specific recombinant enzyme: laronidase for MPS type 1 S and idursulfase for MPS type 2. However, ERT is often associated with infusion-related hypersensitivity reactions (HR) like urticaria, skin rash, flushing, wheezing, and anaphylaxis [2, 3]. 

In MPS patients with HR history, rapid desensitization (RD) can be a valid approach to avoid ERT discontinuation [4]. RD induces temporary tolerance with slow administration (about 6 h) of the offending medication and is performed for both IgE- and non-IgE-mediated HR [5]. Additionally, subcutaneous allergen immunotherapy (AIT), used for Hymenoptera venom-related allergies to induce long-term immune tolerance, includes an induction phase and a maintenance phase, in which a fixed dose of allergen is administered at longer intervals (every 4 weeks). This procedure has been successfully used with protein drugs like laronidase, idursulfase, and trastuzumab for IgE-mediated HR [6, 7]. Recently, omalizumab, an anti-IgE monoclonal antibody, proved to be a valid option in two MPS type 4 A patients who experienced HR to Elosulfase α and were resistant to desensitization approach [8, 9]. This drug acts by binding IgE and prevents its attaching to FceRI (high-affinity IgE receptor), thereby reducing the amount of free IgE that is available to trigger the allergic cascade. Currently, omalizumab is indicated for severe asthma, chronic spontaneous urticaria and chronic rhinosinusitis with nasal polyps [10]. 

We present findings on two-year follow-up and management about 2 MPS patients (Patient 2 and Patient 3) reported in *Spataro et al. 2022*, who initially developed HR to ERT and were subjected to a combined desensitization approach (rapid desensitization plus AIT-like desensitization); the third patient was lost to follow-up for his personal reasons [6]. In this letter, we have maintained the same patient identifiers and sequence as used in the previously published article.

**Fig. 1:**
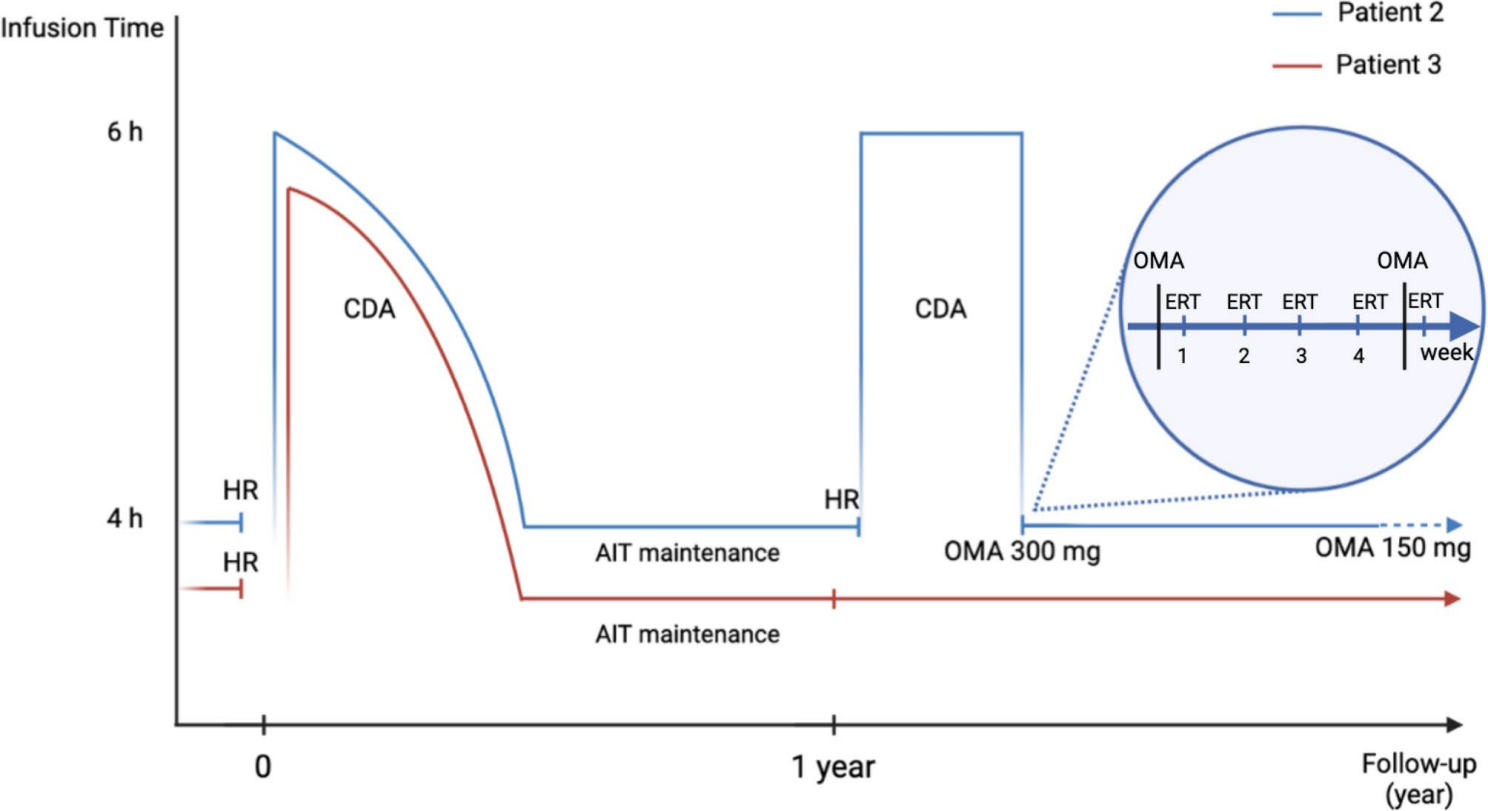
Evolution and management over two years of the two MPS patients allergic to ERT. AIT, allergen immunotherapy; CDA, combined desensitization approach; HR, hypersensitivity reaction; OMA, omalizumab. The blue line represents Patient 2. The red line represents Patient 3

Patient 2 is the 35-year-old man who was diagnosed with MPS type 1 S at age 13 and was safely treated with laronidase administration since July 2021, when he developed diffuse urticaria and lip angioedema during laronidase infusion resulting in drug suspension. The patient was then referred to our Allergy Unit and skin tests with laronidase were performed yielding a positive result and was successfully desensitized with the combined approach: 3-bag, 12-step RD plus a subcutaneous 11-step AIT-like desensitization protocol. After completion of the combined approach, we gradually reduce the laronidase infusion time to reach a standard infusion schedule with only chlorphenamine 10 mg as premedication; simultaneously, we administered laronidase subcutaneously, monthly, as for the classic AIT. Under these conditions, the standard protocol had been performed for 13 months without any HR until December 2022, when he developed generalized urticaria, face angioedema and chest constriction. Then, skin tests were performed again with positive results at 1:100 and 1:10 IDT dilutions (average wheal diameter: 9 and 10.5 mm, respectively). The same combined desensitization approach was begun immediately, but this time unsuccessfully. Thus, we administered omalizumab at the dose of 300 mg two days before the subsequent infusion. Surprisingly, the patient immediately tolerated the subsequent 4 weekly laronidase infusions through standard protocol. Due to the observed benefits, omalizumab 300 mg was administered every 4 weeks (Fig. 1). Moreover, after 6 months, omalizumab was tapered to 150 mg monthly according to the absence of HR occurrence. Under these conditions, the patient is still tolerating ERT.

Patient 3 is the 9-year-old child who was diagnosed with MPS type 2 in 2017 and started the weekly ERT with idursulfase till July 2021, when he developed diffuse urticaria during the last minutes of infusion. In December 2021, the patient was referred to our Allergy Clinic, and an allergy workup with skin tests for idursulfase was carried out with positive test results. Thus, a 3-bag, 12-step RD and, at the same time, the AIT-like desensitization was started. This combined approach allowed the patient to reduce infusion time to a standard infusion protocol with oral cetirizine 7.5 mg as premedication; simultaneously, a monthly administration of the AIT-like desensitization maintenance dose was maintained, subcutaneously. As result, after one year, no HR occurred. Then, we decided to subject the patient to an allergy reevaluation by skin tests with idursulfase (January 2023): both SPT and IDT deemed negative results. AIT-like desensitization was stopped given the patient achieved complete desensitization to idursulfase. The patient is still taking ERT without HR occurrence.

Figure 1 shows a summary of the progression of allergic reactions and their management in the two patients.

In conclusion, ERT represents the only available treatment for MPS, but HR may occur precluding its continuation. Thus, while RD can be a valid approach for the immediate restoration ERT, AIT-like desensitization could be effective in inducing a longer tolerance.

In this experience, the combined desensitization approach proved effective in both cases for at least one year. While Patient 2 lost immune tolerance after 13 months, requiring omalizumab administration, in Patient 3 the combined approach led to complete desensitization, as evidenced by negative skin tests.

This study aims to offer guidance for MPS patients who develop HR to ERT, with the goal of optimizing management and preventing therapy discontinuation. The management algorithm was previously detailed in Spataro et al. 2023 [4]. For patients unable to resume the standard protocol despite RD or a combined approach, omalizumab has shown effectiveness. Here, we propose a practical management strategy for using this monoclonal antibody in similar cases: (i) a monthly omalizumab dose of 300 mg (for adults); (ii) after 6 months, a 50% dose reduction following careful reassessment.

Further studies will be essential to evaluate the efficacy and durability of the combined approach over time, and to determine which patients may benefit the most. Additionally, omalizumab may prove valuable in managing more resistant HR cases, as observed with other treatments, such as chemotherapy [11]. 

## Electronic supplementary material

Below is the link to the electronic supplementary material.

Supplementary Material 1

Supplementary Material 2

## Data Availability

Data and materials used in this case report are based on the medical records, imaging studies and pathological findings of our patient. Due to privacy and confidentiality considerations, access to the specific patient data and materials is restricted. Upon reasonable request, anonymized and de-identified data may be made available for research purposes, in compliance with institutional policies and ethical guidelines.

## Declarations

### Signature

Federico Spataro, Department of Precision and Regenerative Medicine and Jonic Area, Internal Medicine and Allergology Unit, University of Bari Aldo Moro, Italy

## Abbreviations

AIT-like: Allergen immunotherapy-like
ERT: Enzyme replacement therapy
HR: Hypersensitivity reactions
IDT: Intradermal test
MPS: Mucopolysaccharidosis
RD: Rapid desensitization
SPT: Skin prick test
