# Supplementary material for: Two-year follow-up after drug desensitization in mucopolysaccharidosis
Source: Orphanet J Rare Dis. 2024 Dec 27;19:491. doi: 10.1186/s13023-024-03516-z (PMC11673970; doi:10.1186/s13023-024-03516-z)
Supplement: Supplementary file 2 — Supplementary Material 2 [file 13023_2024_3516_MOESM2_ESM.docx]

**ANSWER TO REVIEWER**

Comment to the Author.

The article is now much improved. I have no major criticisms nor suggestions. The authors have replaced the original Figure 1, which described the algorithm for desensitization and was also published elsewhere, with Figure 2. This figure is reworked graphically, but, as far as treatment algorithm is concerned, is essentially the same. The only and, in my view minor change is potential tapering of omalizumab dose. I suggest to omit the figure and refer the reader to the published figure. Details of omalizumanb dosage and their changes are well described in the text and do not justify an addition of another figure.

Answer.

Thank you very much for your comment and valuable advice, which helped us improve this article.
We have removed Figure 2 and referred to the management algorithm already published in the previous study (Spataro et al., 2023) [4].

Thank you again for your thoughtful feedback.
