# Supplementary material for: Two-year follow-up after drug desensitization in mucopolysaccharidosis
Source: Orphanet J Rare Dis. 2024 Dec 27;19:491. doi: 10.1186/s13023-024-03516-z (PMC11673970; doi:10.1186/s13023-024-03516-z)

Bari, March/6th/2024

Francesc Palau, MD, PhD

Editor In-Chief

Orphanet Journal of Rare Diseases

Dear Professor Francesc Palau,

Dear Editors of Lysosomal storage diseases Section,

Hereby we submit the article "TWO-YEAR FOLLOW-UP AFTER DRUG DESENSENSITIZATION IN MUCOPOLYSACCHARIDOSIS" by Federico Spataro, Roberto Ria, Nada Chaoul, Angelo Vacca, Danilo Di Bona, Attilio Di Girolamo, Luigi Macchia to *Orphanet Journal of Rare Diseases*.

This Letter serves as a natural extension of the prior article entitled "Novel approach to idursulfase and laronidase desensitization in type 2 and type 1 S mucopolysaccharidosis (MPS)" published in your estimated *Orphanet Journal of Rare Diseases* (November 2022).

Best regards,

Federico Spataro, MD

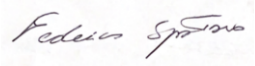

Supplement: Supplementary file 1 — Supplementary Material 1 [file 13023_2024_3516_MOESM1_ESM.pdf]
